# Elevated Blood Lead Concentrations in Essential Tremor: A Case–Control Study in Mersin, Turkey

**DOI:** 10.1289/ehp.10352

**Published:** 2007-08-01

**Authors:** Okan Dogu, Elan D. Louis, Lulufer Tamer, Ozgur Unal, Arda Yilmaz, Hakan Kaleagasi

**Affiliations:** 1 Department of Neurology, Faculty of Medicine, Mersin University, Mersin, Turkey; 2 G.H. Sergievsky Center; 3 Department of Neurology and; 4 Taub Institute for Research on Alzheimer’s Disease and the Aging Brain, College of Physicians and Surgeons, Columbia University, New York, New York, USA; 5 Department of Epidemiology, Mailman School of Public Health, Columbia University, New York, New York, USA; 6 Department of Biochemistry, Faculty of Medicine, Mersin University, Mersin, Turkey

**Keywords:** epidemiology, essential tremor, etiology, lead, neurology, toxicants

## Abstract

**Background:**

Essential tremor (ET) is one of the most common neurologic disorders. Aside from underlying susceptibility genes, recent studies have also begun to focus on environmental toxic factors. Yet there remains a paucity of information on such factors, making studies of environmental factors important. A recent study in New York City found blood lead concentrations to be elevated in ET cases compared with matched controls. Chronic exposure to lead produces cerebellar damage, and this could predispose individuals to develop ET.

**Objective:**

The aim of this study was to determine whether the elevation in blood lead concentrations observed in a single study in New York was similarly present in ET cases sampled from a completely different geographic region.

**Methods:**

Blood lead concentrations were measured in 105 ET cases and 105 controls at Mersin University, Mersin, Turkey.

**Results:**

The median blood lead concentration was 2.7 μg/dL in ET cases compared with 1.5 μg/dL in controls (*p* < 0.001). In an unadjusted logistic regression model, blood lead concentration was associated with diagnosis: odds ratio (OR) = 4.01; 95% confidence interval (CI), 2.53–6.37; *p* < 0.001 (i.e., each 1-μg/dL increase in blood lead concentration was associated with a 4-fold increased odds of ET). This association was more robust when cases were compared with a subsample of controls who did not share the same home environment (OR = 8.13; 95% CI, 3.05–21.65; *p* < 0.001). In adjusted models, results were similar.

**Conclusions:**

These data replicate those of a previous study in New York and demonstrate an association between the environmental toxicant lead and a common neurologic disorder.

Essential tremor (ET) is very common, with a prevalence of 4% in the adult population over 39 years of age and 8.7% in adults over 79 years of age (Dogu et al. 2003). Genetic factors play a sizable role in disease etiology, and susceptibility loci have been identified on chromosomes 3q13, 2p22, and 6p (Deng et al. 2005; Higgins et al. 1998; Shatunov et al. 2006). Although pair-wise concordance in monozygotic twins is high (60–93%) (Lorenz et al. 2004; Tanner et al. 2001), it is not 100%, suggesting an etiologic role for non-genetic factors in some ET cases (Louis 2001). Several case–control studies have examined putative environmental factors, including pesticides (Louis et al. 2006a; Salemi et al. 1998), manganese and organic solvents (Louis et al. 2004), β-carboline alkaloids (Louis et al. 2005c), and lead (Louis et al. 2003, 2005a). In a recent case–control study in New York, New York, blood lead concentrations were elevated in ET cases compared with matched controls (Louis et al. 2003, 2005a). Lead is a ubiquitous toxicant (Louis et al. 2003, Schroeder and Tipton 1968). Laboratory animals and humans exposed to high levels of lead develop prominent action tremor (Booze et al. 1983; Coulehan et al. 1983; Goldings and Stewart 1982; Seshia et al. 1978; Valpey et al. 1978; Young et al. 1977), with destruction of cerebellar Purkinje cells being a major feature of the pathology of lead toxicity (Valpey et al. 1978). This is of particular interest, given recent literature demonstrating mild degenerative cerebellar pathology in ET cases (Louis et al. 2006b, 2006c). It is conceivable that chronic exposure to lead could produce cerebellar damage that then predisposes individuals to develop ET. In this case–control study, ET cases were sampled in the Mersin Province, Turkey, to determine whether the elevation in blood lead concentrations observed in New York was similar to that present in ET cases sampled from a completely different geographic region. An interesting feature of the study design is that ET cases were compared both to spouse controls (who shared the same home environment) and to nonspouse controls (who did not share the same home environment).

## Methods

### Selection of participants

In the Mersin University Hospital Movement Disorder Unit database, 250 ET cases are registered. Each case had a unique registration code and received a diagnosis of ET from their treating neurologist in that unit based on the presence of moderate amplitude action tremor of the arms or head tremor in the absence of other etiologies, such as Parkinson disease. Sample size calculations necessitated 105 cases and 105 controls. Cases who had a final registration code digit of 0, 3, 6, or 9 were first selected for enrollment, resulting in 80 potential enrollees. Then, cases with a final digit code of 2, 5, or 7 were selected, resulting in additional potential enrollees (total *n* = 105). Once selected, cases were asked to enroll in a study of “environmental risk factors for tremor.” Ninety-two (87.6%) cases agreed to be enrolled and 13 declined enrollment; 13 replacement cases were selected based on their digit code, bringing the number of cases up to 105. Most controls were the spouses of the cases [*n* = 69 (65.7%) of 105]. When spouses were not available (18 spouses had died and 18 either refused or could not come to the university), a relative was then selected who lived in the same district in Mersin and was closest in age to the case (nonspouse controls; *n* = 36). Eleven (10.5%) controls declined enrollment, so additional controls had to be selected to obtain the targeted number of 105 controls. Before enrollment, the Mersin University Institutional Review Board approved all study procedures, and written informed consent was obtained at the time of enrollment. Enrollment began on 12 February 2003 and ended on 15 December 2004.

### Demographic and medical history

Once enrolled, all participants were evaluated in person by a neurologist (O.U.), who administered clinical questionnaires and performed a videotaped examination. Data were collected on age, sex, education, cigarette smoking (yes vs. no), cigarette pack-years, ethanol use (yes vs. no), and medication use. ET cases were asked whether they had a first-degree relative with ET. Current occupation was coded into 10 categories (Table 1). Data on past occupation or occupation at the time of diagnosis were not collected.

### Videotaped examination

For all participants, the tester videotaped a tremor examination that included one test to elicit postural tremor (sustained arm extension) and five tests to elicit kinetic tremor (Louis et al. 2003, 2005a). Each of the six tests was performed with each arm (12 tests total) (Louis et al. 2003, 2005a). Each videotape was reviewed by the senior investigator (O.D.), who rated tremor during each of the 12 tests using a 0–3 scale [total tremor score = 0–36 (maximum)] (Louis et al. 2003, 2005a). These ratings were performed blinded to data on blood lead concentrations. O.D. was formally trained to rate tremor using a published teaching videotape (Louis et al. 2001) that included an educational section and a self-assessment section (ratings of 20 items); his 20 ratings demonstrated substantive agreement with published ratings (weighted kappa statistic = 0.81), indicating that his ratings were in agreement with standardized, published ratings of tremor. O.D. also confirmed the diagnosis of ET using published diagnostic criteria [moderate or greater amplitude tremor (tremor rating ≥ 2) during three or more activities or a head tremor in the absence of competing diagnoses such as Parkinson disease] (Louis et al. 1997).

### Determination of blood lead concentrations

On the same day as the clinical questionnaires and videotaped examination, 10-mL blood samples were collected in lead-free stoppered pyrex tubes containing 100 units of heparin. Six milliliters of this heparinized blood was hemolysed with Triton X-100 (octylphenoxypolyethoxyethanol; Sigma 092K0172; Sigma Chemical Co., St. Louis, MO, USA) and analyzed according to the method described by Zinterhofer et al. (1971) using a UNICAM 929 atomic absorption spectrophotometer (UNICAM, Portsmouth, NH, USA) in a trace metal–free environment (Laboratory of Biochemistry, Atomic Absorption Unit, Mersin Hospital). These analyses were performed blinded to clinical information. The detection limit for blood lead using these instruments was 0.1 μg/dL. Intersubject and intrasubject coefficients of variation (CVs) for the lead assay were 0.67% and 0.47%, respectively.

### Statistical analyses

Statistical analyses were performed using SPSS, version 13.0 (SPSS Inc., Chicago, IL, USA). Blood lead concentrations were not normally distributed. Each analysis was first performed using log_10_ blood lead and then repeated using blood lead. The results were similar. Results were presented using blood lead because nontransformed data can be expressed in units of micrograms per deciliter, which is a more easily understandable unit of measure. When examining group differences in blood lead concentration, we compared medians using a nonparametric approach (Mann-Whitney test). To assess associations between blood lead concentration and other continuous variables (e.g., total tremor score) we used Spearman’s correlation coefficients. To evaluate differences between categorical variables, chi-square tests were used. To assess group differences in normally distributed continuous variables, we used the Student’s *t*-test.

Logistic regression analyses were performed. *A priori*, the main analysis was to test the association between blood lead concentration (independent variable) and diagnosis (ET case vs. control, dependent variable). We began with an unadjusted model and then individually considered variables that were suspected to confound the lead–diagnosis association or were known to be associated with blood lead concentration (Louis et al. 2003, 2005a). These were age in years, sex, years of education, current cigarette smoker (yes vs. no), pack-years of smoking, and ethanol use (yes vs. no). We did not need to consider race because this was homogeneous in our sample. Because dietary data were not available, we did not consider reported daily consumptions of vitamin C, calcium, and iron. The confounding variables were entered into the final adjusted multivariate logistic regression model (enter approach) if they were associated with the dependent variable in univariate analyses or if prior evidence supported an association with either blood lead concentration or ET (Louis et al. 2003, 2005a).

## Results

### Cases and controls

The study included 105 ET cases and 105 controls (including 69 spouses and 36 nonspouses). ET cases had a mean ± SD total tremor score of 18.0 ± 6.2 and mean disease duration of 9.6 ± 10.4 (range, 1–49) years; 68 (64.8%) reported having a family history of ET (i.e., an affected first-degree relative), 31 (29.5%) had head tremor on examination, and 56 (53.3%) were taking medication to treat tremor. Controls had a mean total tremor score of 1.0 ± 2.9 and none had ET; 8 (7.6%) had a family history of ET. ET cases and control subjects were similar in terms of age and other demographic variables (Table 1). Data on current occupation were available on 104 ET cases and 105 controls (Table 1); a larger proportion of ET cases was retired compared with controls.

Spouse controls and nonspouse controls had marginally different blood lead concentrations, with spouse controls having marginally higher blood lead concentrations than controls who did not share the same home environment with the cases (mean ± SD, 1.64 ± 0.90 μg/dL vs. 1.44 ± 0.35 μg/dL; medians, 1.5 μg/dL vs. 1.3 μg/dL; Mann-Whitney *z* = 1.52; *p* = 0.13). Among controls, blood lead concentration was not significantly associated with age (*r* = −0.04; *p* = 0.69), sex (both medians = 1.5 μg/dL; Mann-Whitney *z* = 0.60; *p* = 0.55), education (*r* = 0.14; *p* = 0.16), cigarette smoking (both medians = 1.5 μg/dL; Mann-Whitney *z* = 0.99; *p* = 0.32), cigarette pack-years (*r* = 0.11; *p* = 0.28), or ethanol use (both medians = 1.5 μg/dL; Mann-Whitney *z* = 0.62; *p* = 0.54). The median blood lead concentration among retired controls (1.3 μg/dL) was marginally lower than that of nonretirees (1.5 μg/dL; Mann-Whitney *z* = 1.6; *p* = 0.10).

Among cases, blood lead concentration was not significantly associated with age (*r* = 0.12; *p* = 0.22), sex (median in males = 3.1 μg/dL; in females = 2.5 μg/dL; Mann-Whitney *z* = 1.33; *p* = 0.19), cigarette smoking (median in smokers = 3.3 μg/dL; in nonsmokers = 2.7 μg/dL; Mann-Whitney *z* = 0.47; *p* = 0.64), cigarette pack-years (*r* = 0.05; *p* = 0.59), or ethanol use (both medians = 2.7 μg/dL; Mann-Whitney *z* = 1.01; *p* = 0.31). Among cases, blood lead concentration was marginally associated with education (*r* = 0.19; *p* = 0.06). The median blood lead concentration was the same in retired and nonretired cases (both medians = 2.7 μg/dL; Mann-Whitney *z* = 0.02; *p* = 0.98).

### Blood lead concentrations

**Figure 1:**
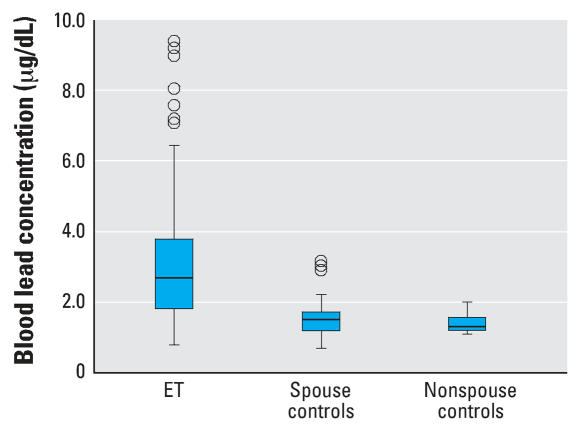
Box plot of blood lead concentrations in ET cases, spouse controls, and nonspouse controls. Horizontal lines indicate the median; boxes represent the interquartile range; whiskers indicate quartiles; and circles indicate outliers.

The median blood lead concentration in ET cases was 2.7 μg/dL compared with 1.5 μg/dL in controls (Mann-Whitney *z* = 8.12; *p* < 0.001). The mean (± SD) blood lead concentrations were 3.2 ± 1.9 μg/dL (range = 0.8–9.4 μg/dL) for cases and 1.6 ± 0.8 μg/dL (range = 0.7–8.0 μg/dL) for controls. The median blood lead concentration in ET cases (2.7 μg/dL) was higher than that of spouse controls (1.5 μg/dL; Mann-Whitney *z* = 6.91; *p* < 0.001) and non-spouse controls (1.3 μg/dL; Mann-Whitney *z* = 6.25; *p* < 0.001) (Figure 1).

In an unadjusted logistic regression model, blood lead concentration was associated with diagnosis (control vs. ET case): odds ratio (OR) = 4.01; 95% confidence interval (CI), 2.53–6.37; *p* < 0.001 (i.e., each 1-μg/dL increase in blood lead concentration was associated with a 4-fold increased odds of ET) (Table 2). In a series of six logistic regression models, we adjusted for a variety of covariates (in each model we included blood lead concentration and one covariate); in these analyses, the association between blood lead concentration and ET remained robust (Table 2). In a model in which we simultaneously adjusted for age, sex, education, cigarette smoking (yes vs. no), cigarette pack-years, and ethanol use (yes vs. no.), blood lead concentration was associated with diagnosis (OR = 4.19; 95% CI, 2.59–6.78; *p* < 0.001). Adding current occupation (retired vs. nonretired) to the model did not change the results. This association was more robust when ET cases were compared with nonspouse controls (unadjusted OR = 8.13; 95% CI, 3.05–21.65; *p* < 0.001; and adjusted OR = 9.87; 95% CI, 2.49–39.22; *p* = 0.001) than with spouse controls (unadjusted OR = 3.28; 95% CI, 2.06–5.23; *p* < 0.001; and adjusted OR = 3.39; 95% CI, 1.96–5.87; *p* < 0.001).

We found a correlation between tremor severity (total tremor score) and blood lead concentration (*r* = 0.48; *p* < 0.001) in the entire sample. This correlation was likely a reflection of the difference between cases and controls because it was not present in analyses restricted to ET cases (*r* = −0.12; *p* = 0.23). The correlation between blood lead concentration and tremor duration in ET cases was not significant (*r* = 0.17; *p* = 0.09), especially after adjusting for age (*r* = 0.05; *p* = 0.62). ET cases were stratified based on whether each case currently took medication for ET, had a family history of ET, or had head tremor, but there were no differences with respect to median blood lead concentrations.

## Discussion

Given the paucity of information on risk factors for ET, studies of environmental factors are important. In a previous study, blood lead concentrations were elevated in ET cases compared with matched controls. Now, in this case–control study in Mersin, Turkey, we found that blood lead concentrations were approximately doubled in ET cases compared with their counterparts without ET. This association between higher blood lead concentration and the diagnosed ET persisted after adjusting for confounding variables. This association was particularly robust when ET cases were compared with controls who did not share their same home environment (i.e., nonspouse controls); in these analyses, each 1-μg/dL increase in blood lead concentration was associated with an 8-fold increased odds of having ET.

The present results are similar to those of the New York study (Louis et al. 2003, 2005a) in the sense that ET was associated with a modest yet significant increase in blood lead concentration. The higher ORs in the present study are probably due to the fact that blood lead concentrations were slightly lower in the Mersin controls than the New York controls (Louis et al. 2003, 2005a).

Blood lead concentrations in ET cases were higher than those observed in both types of controls. These data suggest that the increased blood lead concentration in these ET cases is robust. It is not clear whether the difference between cases and controls is due to increased environmental exposure or genetic differences in lead metabolism. Previous work in New York suggests that lead metabolism may be altered in ET cases compared with control subjects; in that study (Louis et al. 2005a), the odds of ET were greatly elevated (OR = 80.29; 95% CI, 3.08–2096.36; *p* = 0.008) in individuals with both a δ-amino-levulinic acid dehydratase-2 allele and an elevated blood lead concentration.

Although the blood lead concentration differed between ET cases and controls, as in the previous study in New York (Louis et al. 2003, 2005a), the concentrations in these study participants were relatively low. However, evidence suggests that low levels may be associated with adverse health effects. In a report of 141 men taking part in a normative aging study, with mean blood lead concentrations of 5.5 μg/dL (Payton et al. 1998), higher concentrations of blood and bone lead were associated with poorer performance on cognitive tests. In a study of older women (Muldoon et al. 1996), blood lead levels as low as 8 μg/dL were significantly associated with poorer cognitive function as measured by certain neuropsychologic tests. Lead exposure may also be associated with other neurologic diseases, although the number of such studies is small. In one study (Kamel et al. 2005), amyotrophic lateral sclerosis was associated with self-reported occupational lead exposure and blood and bone lead levels. In another study (Coon et al. 2006), risk of Parkinson disease was elevated among individuals in the highest quartile for lifetime lead exposure.

Humans may be exposed both to inorganic and organic forms of lead from a variety of occupational and nonoccupational sources (Coulehan et al. 1983; Winegar et al. 1997). In humans and laboratory animals, lead exposure may lead to acute and chronic progressive disorders in which action tremor is a prominent feature (Booze et al. 1983; Coulehan et al. 1983; Goldings and Stewart 1982; Seshia et al. 1978; Valpey et al. 1978; Young et al. 1977). Lead toxicity causes cerebellar pathology. Rat pups fed a diet containing 4% lead acetate demonstrated changes in the topology of Purkinje cell dendritic trees due to a change in Purkinje cell metabolism (McConnell and Berry 1979). Perinatal exposure to inorganic lead results in degenerative changes in Purkinje cells in the rabbit cerebellum (Walsh and Tilson 1984). An autopsy study of humans with chronic organic lead exposure revealed severe destruction of cerebellar Purkinje cells (Valpey et al. 1978). Multiple lines of evidence suggest that the cerebellum is abnormal in ET, including clinical, imaging, electrophysiologic, and recent pathologic studies (Louis et al. 2006b, 2006c; Stolze et al. 2000; Wills et al. 1994). Given the cerebellar toxicity of lead (Valpey et al. 1978; McConnell and Berry 1979; Walsh and Tilson 1984) and the emerging links between ET and cerebellar degeneration (Louis et al. 2006b, 2006c; Stolze et al. 2000; Wills et al. 1994), it seems plausible that the association between the two could be robust.

The present analyses were cross-sectional. The data do not directly address the issue of whether higher blood lead concentrations preceded or followed the diagnosis of ET. Prospective studies are needed to assess causality by assessing whether a higher pre-disease blood lead concentration is associated with an increased risk of developing incident ET. One possibility is that higher blood lead concentrations result in ET, with a possible mechanism being lead-induced cerebellar damage. Another possibility is the converse, namely, that having ET results in higher blood lead concentrations, although the potential mechanisms for such a relationship are not readily apparent. A final possibility is that some common underlying factor (e.g., a genetic predisposition) leads both to ET and to elevated blood lead concentrations.

Our study had additional limitations. First, our occupational assessment was limited to current occupation rather than lifetime occupation. However, in the previous report (Louis et al. 2003), which used lifetime occupational histories to estimate the probability of lifetime occupational exposures to lead, no association was found between ET and occupations with lead exposure. Also, in Mersin, the place of residence and occupation are relatively stable compared with areas where residents are more highly educated, mobile, and wealthier; therefore current occupation in the present study likely reflects lifetime occupation. Second, dietary data were not available, so we were not able to adjust for reported daily consumptions of vitamin C, calcium, and iron, which have been reported to be associated with higher blood lead levels (Louis et al. 2003, 2005a). However, in our previous study (Louis et al. 2003) in which we adjusted for these dietary factors, the case–control differences in blood lead concentration remained robust. Also, in a published comparison of several hundred ET cases and control subjects (Louis et al. 2005b), daily dietary consumption of vitamin C, calcium, and iron did not differ. However, the effect of these unmeasured confounders on our ORs is not known. Third, we assessed blood lead concentrations rather than bone lead concentrations. Bone lead concentrations are a better measure of cumulative exposure to lead, although there is a correlation between the two in “steady-state” exposure (Cheng et al. 1998). Our use of blood lead as a measure of lead exposure might not have optimized our ability to detect an association between lead exposure and ET. Fourth, our sample was from a tertiary referral hospital that contains the only movement disorder unit in the region. Hence, the sample was not population based, so we cannot necessarily generalize our results to ET cases ascertained directly from the population of Mersin. Although it is possible that selection bias accounted for our observations, this is unlikely; the selection of ET cases was not predicated on their blood lead concentrations, which were assessed only after they had visited the Movement Disorder Unit. Finally, although the use of spouses as controls can lead to limitations (in this setting because they shared the same home environment), this would have biased our results toward rather than away from the null hypothesis. Furthermore, we also enrolled a sample of nonspouse controls and demonstrated a difference between cases and controls as well. In spite of these limitations, this is one of only two studies to explore differences in blood lead concentrations between ET cases and controls.

In summary, data in the present study replicate those of a previous study conducted in New York (Louis et al. 2003, 2005a) and demonstrate an association between the environmental toxicant lead and a common neurologic disease, ET. In doing so, they provide additional support for the notion that environmental factors could be involved in disease etiology. Prospective studies are needed to assess causality by assessing whether a higher predisease blood lead concentration is associated with an increased risk of developing incident ET.

## Figures and Tables

**Table 1:** Demographic characteristics of ET cases and controls.

		Controls		
Characteristic	ET cases (*n* = 105)	All (*n* = 105)	Spouse (*n* = 69)	Nonspouse (*n* = 36)
Age (years)	52.9 ± 18.6	50.7 ± 13.7	50.9 ± 12.5	50.3 ± 15.9
Female sex	42 (40.0)	42 (40.0)	36 (52.2)	6 (16.7)*
Education (years)	8.7 ± 4.1	8.5 ± 3.9	8.4 ± 3.9	8.7 ± 4.0
Current cigarette smoker	28 (26.7)	35 (33.3)	19 (27.5)	16 (44.4)*
Cigarette pack-years	3.7 ± 7.5	4.8 ± 7.9	3.8 ± 7.7	6.6 ± 8.2
Ethanol user	33 (31.4)	24 (22.9)	11 (15.9)*	13 (36.1)
Current occupation				
Housewife	28 (26.9)	33 (31.4)	25 (36.2)	8 (22.2)
Officer	8 (7.7)	12 (11.4)	9 (13.0)	3 (8.3)
Farmer	0 (0.0)	4 (3.8)	0 (0.0)	4 (11.1)
Industrial worker	2 (1.9)	1 (1.0)	0 (0.0)	1 (2.8)
Student	6 (5.8)	2 (1.9)	2 (2.9)	0 (0.0)
Retailer	6 (5.8)	14 (13.3)	6 (8.7)	8 (22.2)
Unemployed	2 (1.9)	3 (2.9)	2 (2.9)	1 (2.8)
Retired	46 (44.2)	30 (28.6)*	21 (30.4)	9 (25.0)*
Teacher	4 (3.8)	4 (3.8)	3 (4.3)	1 (2.8)
Health care	2 (1.9)	2 (1.9)	1 (1.4)	1 (2.8)

Values shown are mean ± SD or no. (%).

* *p* < 0.05 compared with ET cases.

**Table 2:** Logistic regression models.

Model	Variables in model	OR (95% CI)	*p-*Value for each variable in the model
1	Blood lead concentration	4.01 (2.53–6.37)	< 0.001
2	Blood lead concentration	4.00 (2.51–6.37)	< 0.001
	Age in years	1.001 (0.98–1.02)	0.94
3	Blood lead concentration	4.02 (2.53–6.37)	< 0.001
	Female sex	1.14 (0.59–2.21)	0.70
4	Blood lead concentration	4.12 (2.58–6.60)	< 0.001
	Education (years)	0.96 (0.88–1.04)	0.31
5	Blood lead concentration	4.10 (2.57–6.54)	< 0.001
	Current cigarette smoker	0.60 (0.29–1.26)	0.18
6	Blood lead concentration	4.09 (2.57–6.52)	< 0.001
	Cigarette pack-years	0.97 (0.93–1.02)	0.18
7	Blood lead concentration	3.97 (2.50–6.30)	< 0.001
	Ethanol user	1.23 (0.59–2.59)	0.58
